# Three-dimensional reconstruction for coherent diffraction patterns obtained by XFEL

**DOI:** 10.1107/S1600577517007767

**Published:** 2017-06-14

**Authors:** Miki Nakano, Osamu Miyashita, Slavica Jonic, Changyong Song, Daewoong Nam, Yasumasa Joti, Florence Tama

**Affiliations:** a Advanced Institute of Computational Science, RIKEN, 6-7-1 Minatojima-minami-machi, Chuo-ku, Kobe, Hyogo 650-0047, Japan; b IMPMC, Sorbonne Universités – CNRS UMR 7590, UPMC Univ Paris 6, MNHN, IRD UMR 206, Paris 75005, France; c Department of Physics, Pohang University of Science and Technology (POSTECH), Pohang 790-784, Republic of Korea; d XFEL Utilization Division, Japan Synchrotron Radiation Research Institute (JASRI), 1-1-1 Kouto, Sayo-cho, Sayo-gun, Hyogo 679-5198, Japan; e Department of Physics, Graduate School of Science, Nagoya University, Furo-cho, Chikusa-ku, Nagoya, Aichi 464-8602, Japan; f Institute of Transformative Bio-Molecules, Nagoya University, Furo-cho, Chikusa-ku, Nagoya, Aichi 464-8602, Japan

**Keywords:** three-dimensional reconstruction, single-particle analysis, X-ray free-electron laser, coherent X-ray diffraction imaging

## Abstract

The three-dimensional (3D) structural analysis of single particles using an X-ray free-electron laser (XFEL) is a new structural biology technique that enables observations of molecules that are difficult to crystallize, such as flexible biomolecular complexes and living tissue in the state close to physiological conditions. In order to restore the 3D structure from the diffraction patterns obtained by the XFEL, computational algorithms are necessary as the orientation of the incident beam with respect to the sample needs to be estimated. A program package for XFEL single-particle analysis based on the *Xmipp* software package, that is commonly used for image processing in 3D cryo-electron microscopy, has been developed. The reconstruction program has been tested using diffraction patterns of an aerosol nanoparticle obtained by tomographic coherent X-ray diffraction microscopy.

## Abstract 2

The developed reconstruction method can successfully identify the orientations of coherent X-ray diffraction patterns of an aerosol nanoparticle.

## 1. Introduction   

Biological molecules, such as proteins, nucleic acids and their complexes, are responsible for many vital cellular functions, including gene transcription and protein synthesis, and their dysfunctions result in severe diseases (Watson & Crick, 1953 ▸; Aloy & Russell, 2002 ▸; Berman *et al.*, 2003 ▸). Information on the structure and dynamics of biomolecules and biomolecular complexes is helpful for understanding their functional mechanisms, and X-ray crystallography is currently the most widely used technique to determine the three-dimensional (3D) structure of biomolecules (Drenth, 2007 ▸; Rupp, 2009 ▸). However, this technique requires molecules to be crystallized, and it is difficult to apply to insoluble molecules or intrinsically disordered proteins. Although cryo-electron microscopy (cryo-EM) does not require crystallization and can observe heterogeneous samples (Kühlbrandt, 2014 ▸), applications to observe the inner structure of thick objects (more than 500 nm) can be challenging due to multiple-scattering events (Cheng *et al.*, 2015 ▸).

Single-particle imaging using femtosecond X-ray pulses from X-ray free-electron lasers (XFELs) could allow the inner structure of biological molecules to be observed in a state close to nature without crystallization (Neutze *et al.*, 2000 ▸; Huldt *et al.*, 2003 ▸; Chapman *et al.*, 2006 ▸; Gaffney & Chapman, 2007 ▸). Radiation damage is a serious problem in high-resolution microscopy as it reduces the spatial resolution of the molecular structure (Henderson, 1995 ▸; Kirz *et al.*, 1995 ▸; Cheng *et al.*, 2015 ▸). XFELs can significantly relax the resolution barrier imposed by radiation damage by recording the diffraction pattern before specimen destruction, due to femtosecond-short pulse duration (Neutze *et al.*, 2000 ▸; Chapman *et al.*, 2011 ▸; Hirata *et al.*, 2014 ▸; Suga *et al.*, 2014 ▸). XFEL experimental data are becoming increasingly available and several low-resolution structures from single-particle approaches have been reported (Seibert *et al.*, 2011 ▸; Gallagher-Jones *et al.*, 2014 ▸; Kimura *et al.*, 2014 ▸; Xu *et al.*, 2014 ▸; Ekeberg *et al.*, 2015 ▸; Takayama *et al.*, 2015 ▸; van der Schot *et al.*, 2015 ▸). It has also been shown, theoretically, that high-resolution 3D structures could be obtained using millions of diffraction patterns (Loh & Elser, 2009 ▸; Tegze & Bortel, 2012 ▸; Tokuhisa *et al.*, 2012 ▸; Hosseinizadeh *et al.*, 2014 ▸) and that dynamics could be directly extracted from two-dimensional (2D) data (Tokuhisa *et al.*, 2016 ▸).

However, there are still challenging problems in obtaining high-resolution 3D structures of biomolecules from XFEL experimental data. Because the diffraction intensity from biological molecules is too weak, an insufficient photon count is a serious problem, especially at high-wavenumber pixels which determine the resolution in real space. On the other hand, the diffraction intensity at low-wavenumber pixels is too strong and saturates the detection range, which hinders the determination of the overall shape of the system by phase-retrieval procedures. In addition, 3D imaging requires the assembly of diffraction patterns from many identical copies of a reproducible object. Therefore, 2D diffraction images should be obtained from structurally homogeneous samples, but it is generally difficult for sub-micrometer systems, which is the typical target size for XFEL single-particle analysis.

Along with these challenges, one of the critical algorithmic problems to be solved in order to reconstruct 3D structures from diffraction patterns obtained in XFEL experiments is the estimation of the orientation of single particles with respect to the laser beam (three Euler angles) (Loh & Elser, 2009 ▸; Philipp *et al.*, 2012 ▸; Ekeberg *et al.*, 2015 ▸). This is also a common problem in cryo-EM single-particle analysis. Several open-source software packages have been developed to reconstruct 3D molecular structures from cryo-EM projection images (Grigorieff, 2007 ▸; Tang *et al.*, 2007 ▸; Shaikh *et al.*, 2008 ▸; Scheres, 2012 ▸; de la Rosa-Trevín *et al.*, 2013 ▸). There are currently two main strategies for estimating the 3D orientation of particles. One is called projection matching (Penczek *et al.*, 1994 ▸; Sorzano *et al.*, 2004 ▸; Grigorieff, 2007 ▸; Yang & Penczek, 2008 ▸), where the best angular parameters are estimated by finding an image which has a maximum correlation coefficient in the reference library and is used to construct a new volume. Another widely used strategy is the maximum-likelihood-based algorithm (Scheres *et al.*, 2007 ▸; Sigworth *et al.*, 2010 ▸; Scheres, 2012 ▸; Lyumkis *et al.*, 2013 ▸). In these approaches, a number of angular assignments are considered for each target image, and are concurrently used for reconstruction with relative weights based on the similarities between the target image and reference images.

While a cryo-EM electron density image contains structural information in real space, an XFEL diffraction pattern contains structural information in Fourier space and is related to a slice (Ewald sphere) of the 3D diffraction intensity. Thus, 3D reconstruction from XFEL data can be performed using similar procedures to those used in cryo-EM. For instance, ‘slice matching’ can be performed to determine the orientation of the diffraction patterns. One of the algorithms successfully applied for 3D reconstruction from XFEL data is EMC (Ekeberg *et al.*, 2015 ▸), which uses a ‘maximum likelihood’ approach. A ‘maximum cross correlation’ algorithm for 3D reconstruction was previously tested with synthetic data using a large number (8000–100000) of diffraction patterns (Tegze & Bortel, 2012 ▸). Here, we aim to reconstruct a 3D model employing a ‘maximum cross correlation’ algorithm with experimental data and to examine necessary parameter calibrations in detail. Our maximum cross-correlation algorithm is similar to that of Tegze & Bortel, but there are also some differences between the two algorithms as follows: we reconstructed the 3D structure factor amplitude instead of the 3D diffraction intensity by using a weight function based on the Kaiser–Bessel window. A phase recovery procedure is needed to obtain a 3D model in real space from the assembled 3D model in Fourier space (assembled from the diffraction patterns at the determined orientations). However, this task is also not trivial and its result may depend on the choice of the phase recovery algorithm (Sekiguchi *et al.*, 2016 ▸); therefore we here focus on the angular assignment process only.

Our program was implemented on top of *Xmipp*, which is an image-processing software package primarily aimed at single-particle 3D cryo-EM (de la Rosa-Trevín *et al.*, 2013 ▸). The program suite contains many useful tools for image analysis that could be used for analyzing XFEL data. We tested our reconstruction program using experimental diffraction data of an aerosol nanoparticle obtained by tomographic coherent X-ray diffraction microscopy (CXDM) (Miao *et al.*, 2006 ▸; Barty *et al.*, 2008 ▸; Jiang *et al.*, 2010 ▸) instead of data from XFEL. These data are very similar to those from XFEL experiments, the main difference being that XFEL is ‘single-shot’ while X-ray diffraction microscopy allows (weaker but) repeating exposure. The speckle patterns from CXDM were employed to simulate XFEL single-particle data, *i.e.* a set of diffraction patterns obtained from a non-crystalline nanoparticle with different sample orientations. Sample orientations were estimated using the approach demonstrated here, and the assigned angles were later compared with the angles actually used for the data collection. We discuss the choice of parameters and protocols for a successful estimation of the incident beam angles.

## 2. Theory: reconstruction of 3D structure in Fourier space from diffraction patterns   

**Figure 1:**
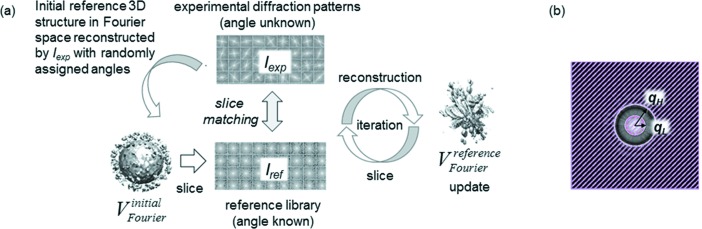
(*a*) Schematic view of our program for volume reconstruction from experimental diffraction patterns. (*b*) Matching region in the diffraction pattern used for calculation of cross correlation. Center and outer regions of the diffraction pattern are masked (filled with pink stripes).

We performed the reconstruction of the structure factor amplitude distribution (hereafter called ‘volume’), from diffraction patterns, based on the ‘slice matching’ protocol (Fig. 1*a* ▸). While others reconstruct 3D diffraction patterns by assembling 2D diffraction patterns in 3D space (Tegze & Bortel, 2012 ▸; Ekeberg *et al.*, 2015 ▸), we here reconstruct 3D amplitudes, because the 3D amplitude can be directly used for phase retrieval. More precisely, we convert 2D diffraction patterns to structure factor amplitude and then assemble into 3D space (comparison results between 3D diffraction amplitude distribution and 3D diffraction intensity are shown in §4). The protocol is as follows:

(i) Create an initial volume, 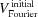. In general, we create 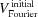 using a set of 2D diffraction patterns with incident beam angles that are randomly assigned. Each 2D diffraction pattern has been centro-symmetrized, and the square roots of the intensities are calculated to obtain structure factor amplitude before calculation. In the first iteration, 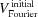 is used as the reference volume, 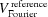.

(ii) Create a reference library of *N*_ref_ diffraction patterns by extracting, using cubic spline interpolation, slices from 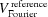 at orientations (angles φ and θ) distributed over a sphere evenly, using a given angular sampling (discretization) step (Bunge & Baumgardner, 1995 ▸). Then, square slice pixel values to obtain the corresponding diffraction patterns.

(iii) Calculate the zero-mean normalized cross correlation, CC, between each experimental diffraction pattern and all reference patterns rotated by angles ψ in plane (with a given angular discretization step) using the following equation,
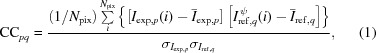
where *I*_exp,*p*_(*i*) and *I*_ref,*q*_(*i*) are the diffraction intensity at pixel *i* of the *p*th experimental and *q*th reference diffraction patterns, respectively (*p* = 1 to *N*_exp_, *q* = 1 to *N*_ref_). *N*_pix_ is the number of pixels in each diffraction pattern. 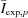 and 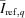 are the average intensities of the *p*th experimental and *q*th reference diffraction patterns, and 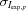 and 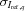 are their standard deviations, respectively. 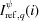 is the diffraction intensity of the *q*th reference pattern rotated by angle ψ in the plane. The reference pattern and ψ resulting in the maximum CC coefficient (CC^max^) are denoted by 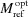 and ψ^opt^, respectively. The incident beam angles φ^opt^ and θ^opt^ used to create 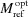 and the in-plane angle ψ^opt^ assigned to the experimental image are the Euler angles set which determines the particle orientation. For the calculation, we apply masks as described below to enhance the sensitivity of the angular assignment.

(iv) Reconstruct a volume, *V*_Fourier_, using the experimental images with the Euler angles assigned as described above. The diffraction amplitude at voxel *k* in the reconstructed volume, *A*(*k*), is obtained as the weighted average of the square roots of the diffraction intensities, 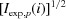, in the experimental diffraction images [equation (2)]. To calculate *A*(*k*), a weight function based on the Kaiser–Bessel window is used (Lewitt, 1990 ▸; Abrishami *et al.*, 2015 ▸), which depends on the distance *d*_*kj*_ between the position *k* and *j* within *V*_Fourier_: *k* is the center position of the voxel *k* and *j* is the position of the pixel *i* within the 2D diffraction image, *p*, in a 3D volume [equation (3)],
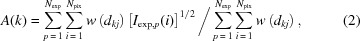


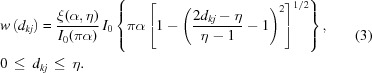

*I*_0_ is the zeroth-order modified Bessel function, η is the maximum radius for interpolation, and α is a variable which determines the decreasing rate of *w*(*d*_*kj*_). ξ(α,η) is the normalization factor determined by α and η. With a large η, the diffraction intensity would be interpolated using the pixels farther in the mapped position in the reconstructed volume. With a large α, the weight for the interpolation would be decreased quickly as *d*_*kj*_ increases. These parameters need careful calibration for successful reconstructions and are discussed later in detail. Finally, we centro-symmetrized the reconstructed volume in Fourier space.

(v) Update 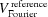 using the volume reconstructed in the previous step. The reference library sampling steps will be made smaller for the refinement of angle assignment in the next iteration. The method allows the correlation coefficient between the experimental diffraction image and the reference diffraction images to be calculated in the vicinity of the currently assigned angles. This is an option of the method that should not be used in the beginning of the iterative assignment process.

(vi) Iterate from (ii) to (v) until convergence of 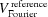. The final 3D structure is denoted as 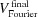.

In the calculation of CC, we excluded the center and outer regions of the diffraction patterns to improve the sensitivity of the matching. The center region is excluded in the calculation since these very large intensity values reduce the sensitivity of the CC calculation. Also, in practice, these diffraction intensities are often too strong to be measured and are thus missing in the data, especially in XFEL applications. In the outer region of diffraction patterns, intensities are usually too weak to be detected and are uncertain because of noise. Therefore, we only calculated CC for the annular regions defined by the inner and outer radii, *q*_L_ and *q*_H_, as shown in Fig. 1(*b*) ▸.

The ‘slice matching’ approach described above [steps (i)–(vi)] was implemented based on a projection matching protocol included in *Xmipp*, which uses a Fourier-space representation of the reference volume for library creation as well as 3D interpolation in Fourier space for 3D reconstruction.

## 3. Results   

### 3.1. Pre-processing of experimental diffraction patterns   

To test our ‘slice matching’ protocol, we used tomographic CXDM data of aerosol nanoparticles. The similarity between CXDM and single-particle XFEL resides in the absence of sample crystallization and in the sample irradiation from different incident beam orientations. However, different diffraction patterns collected in a CXDM experiment correspond to different orientations of the same sample while each single-particle XFEL diffraction pattern corresponds to an orientation of a different sample. Additionally, in tomographic CXDM, the sample is only rotated around one axis (one angle, θ, is known) with respect to the incident beam, whereas all three Euler angles (φ, θ and ψ) are unknown in single-particle analysis using XFELs.

Our final goal is to reconstruct the 3D structure using XFEL experimental diffraction images. The aim of this study is to validate the incident beam angle estimation in our reconstruction program using experimental diffraction images including noise and obtain insight into the calibration of the necessary parameters. Diffraction images obtained by tomographic CXDM are ideal data for our purpose, since incident beam angles with respect to the sample are known (the tilt angles were set experimentally). Pretending that we do not know the incident beam angles, we used our program to estimate the orientation of each diffraction pattern, and compared against the actual orientation. A total of 53 diffraction patterns at various sample orientations (tilting angles from −69° to +69° in 1° to 4° increments in an equal slope scheme) were measured using a CCD camera. The specimen size used in this study was about 1.5 µm. The data were all 3 × 3 binned and resized to 430 pixel × 430 pixel. All diffraction patterns were centro-symmetrized.

**Figure 2:**
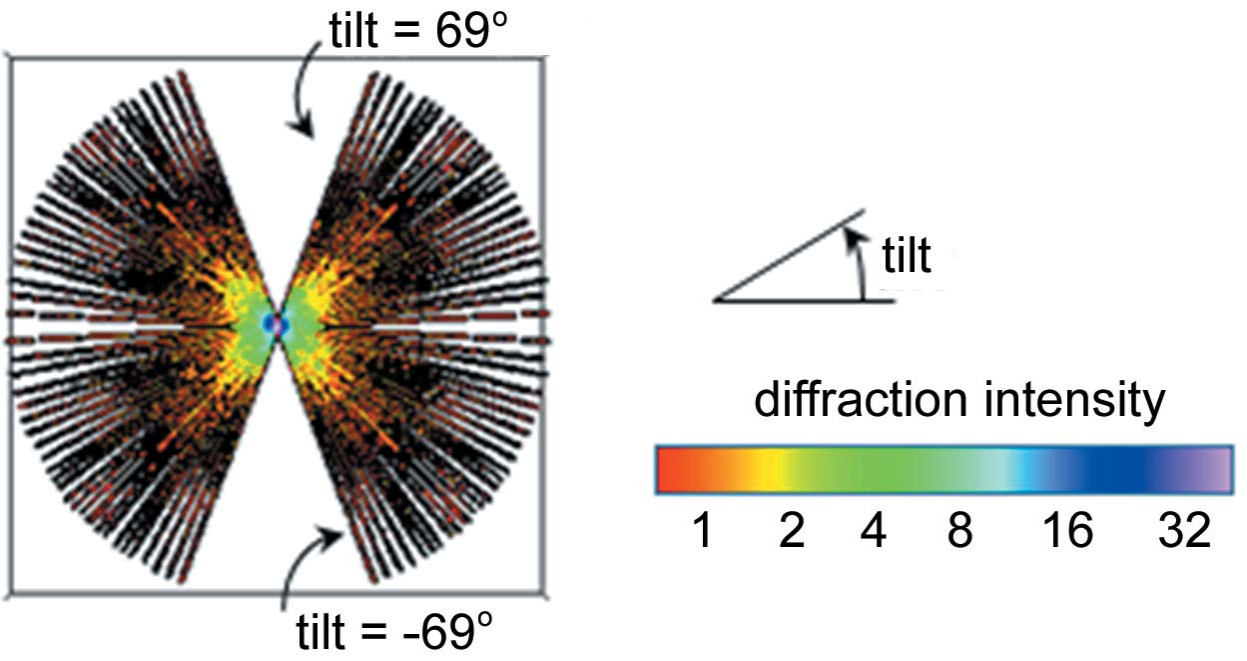
Cross section of the volume 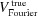 that was assembled from experimental diffraction patterns arranged in 3D Fourier space using the ground-truth angles. The tilt axis is perpendicular to the cross section.

Fig. 2 ▸ shows a cross-section view of the arrangement of the full-size experimental diffraction patterns in 3D Fourier space using the ground-truth incident beam angles, 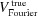, with the interpolation parameters η = 1 pixel and α = 15. The tilt axis is perpendicular to the cross section shown in Fig. 2 ▸. The empty regions in this 3D space are related to the limitations in the maximum tilt angle achievable with the given CXDM apparatus, which is identical to the missing wedge problem in cryo-electron tomography (Lučić *et al.*, 2005 ▸). Also, there is one common line shared by all experimental diffraction patterns.

**Figure 3:**
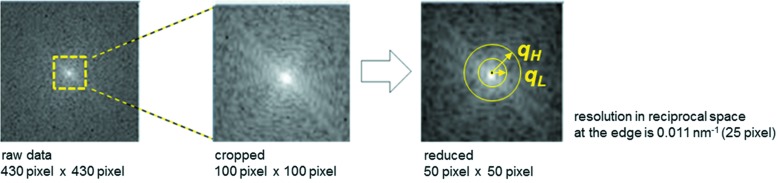
Pre-processing of the experimental diffraction patterns.

Before applying the 3D reconstruction algorithm, we cropped the outer region of the diffraction patterns to remove the pixels which do not practically carry diffraction information (the values of pixels much farther from the central pixel are usually zero or too small to be distinguished from noise). The resolution in reciprocal space at the edge of the cropped image is approximately 0.011 nm^−1^ (= *q*_max_), which corresponds to 91 nm resolution in real space. Furthermore, we reduced the image size from 100 pixel × 100 pixel to 50 pixel × 50 pixel by binning (Fig. 3 ▸). These reductions were necessary in order to cover a sufficient number of voxels to ensure that nearby 2D images in the assembled 3D volume are close enough to detect the consistency (and inconsistency) of the 3D reconstruction. Cropping the outer region of a diffraction pattern corresponds to reducing the resolution in real space, while diffraction pattern binning (reducing the oversampling in Fourier space) corresponds to denoising in Fourier space (without binning, the diffraction intensity fluctuates more due to noise, including Poisson noise). The curvature of the Ewald sphere can be ignored, taking into account the current resolution limit. Regarding searching orientations of diffraction patterns by 2D slice matching of a 3D structure in Fourier space, diffraction pattern cropping is important because the search is focused on the central region that contains information about the object’s shape in real space.

### 3.2. Adjustments of beam intensity variations   

**Figure 4:**
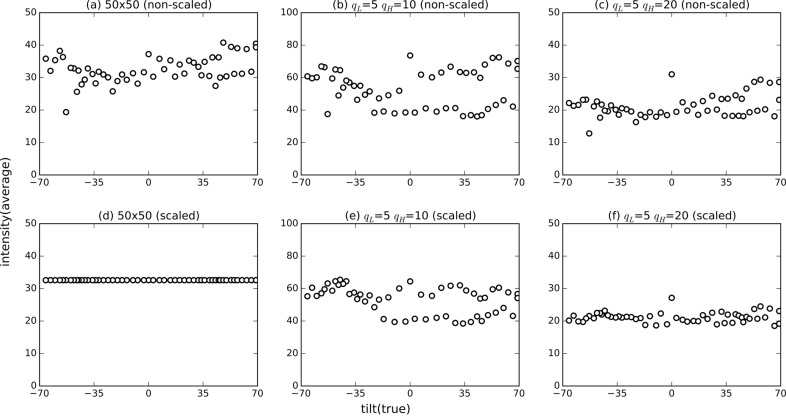
Intensity of each diffraction pattern for non-scaled data [(*a*)–(*c*)] and for scaled data [(*d*)–(*f*)] averaged over the cropped image regions of size reduced to 50 pixels × 50 pixels (*a*,*d*), within annular region *q*_L_ = 5 pixels and *q*_H_ = 10 pixels (*b*,*e*), and within annular region *q*_L_ = 5 pixels and *q*_H_ = 20 pixels (*c*,*f*). The horizontal-axis tilt(true) represents the ground-truth incident beam angles.

To reconstruct 3D structures, variations of beam intensity embedded in the diffraction patterns need to be normalized. Fig. 4(*a*) ▸ shows the diffraction intensities averaged over the cropped region reduced to 50 pixel × 50 pixel. In Fig. 4(*a*) ▸, the average diffraction intensity over all ground-truth tilt angles was 32.59 ± 4.15. It is expected that the average diffraction intensity is not the same for different ground-truth tilt angles and that a continuous change is possible. In this study, we normalized (scaled) the cropped diffraction patterns (50 pixel × 50 pixel) so that the average intensity is the same for all ground-truth tilt angles [Figs. 4(*a*) and 4(*d*) ▸]. Note that, in all scaled diffraction patterns, the sum of diffraction intensities at low wavenumbers (*q*_L_ < 5 pixel) is 95% of the total sum. This scaling smoothens the variations of the intensity averaged over the annular regions defined by *q*_L_ and *q*_H_, as shown in Figs. 4(*b*), 4(*c*), 4(*e*) and 4(*f*) ▸.

### 3.3. Adjustment of the interpolation parameters   

**Figure 5:**
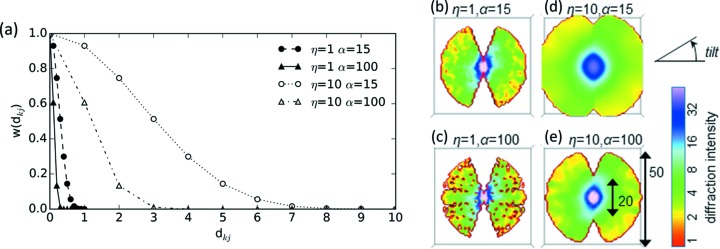
(*a*) Normalized interpolation weight function based on the Kaiser–Bessel window. *d*_*kj*_ is the distance between the center of the voxel *k* within the volume and the pixel *i* within the 2D image mapped to the position in the volume. The values of *w*(*d*_*kj*_) are normalized at the values of *d*_*kj*_ = 0 for each parameter set. (*b*–*e*) Cross sections of the Fourier volumes reconstructed using the pre-processed experimental diffraction patterns, their ground-truth angles and various interpolation parameters (the same cross-section view is shown for four volumes). The tilt axis is perpendicular to the provided cross sections.

To find optimal interpolation parameters for 3D reconstruction, we reconstructed volumes using experimental images (pre-processed as described in the previous sections) and their ground-truth orientations (ground-truth tilt angles), with various interpolation parameters for 3D reconstruction [Figs. 5(*a*)–5(*e*) ▸]. The interpolation radius, η, needs to be sufficiently large to fill the reciprocal space where experimental data are missing. The parameter α controls the relative weight for the interpolation; with a smaller α, data points are more equally counted, regardless of the distance, and the reconstructed volume becomes blurred. The maximum diffraction pattern frequency used for the 3D reconstruction was set to 0.8 *q*_max_ (= 20 pixels) to avoid the protrusion of the intensity outside the volume box when using large values of η.

The volumes reconstructed with the interpolation parameter, η = 1, show discontinuity inside [Figs. 5(*b*) and 5(*c*) ▸]. In the 3D reconstruction algorithm, angles are assigned to maximize the consistency between neighboring slices. With such a small η, each voxel is determined merely by the nearest slice (diffraction pattern); in other words, neighboring slices have no influence on each other, and thus there is no inconsistency to be reduced. Indeed, when angular assignment is started from a set of random angles, convergence is often reached within a few iteration steps without any improvement (results not shown). Thus, we need to use a sufficiently large η. With η = 10, the interpolated 3D volume shows continuity [Figs. 5(*d*) and 5(*e*) ▸], indicating that diffraction patterns sufficiently influence each other in an assembled 3D space.

**Figure 6:**
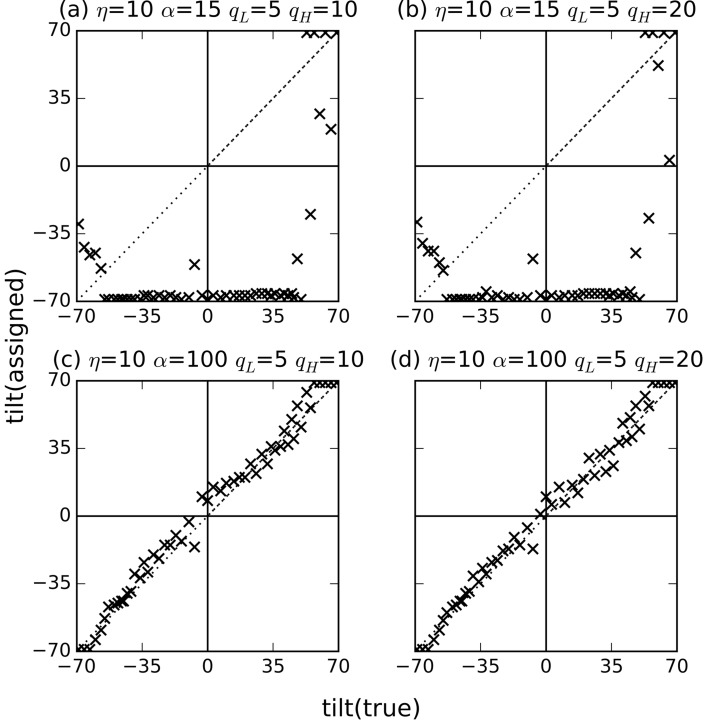
Estimated tilt angles of experimental diffraction patterns using the alignment initiated with ground-truth angles and the 3D reconstruction with various interpolation parameters.

We then optimized other interpolation parameters for angular assignment. We performed iterations starting from the initial reference volumes obtained from images (pre-processed as explained above) with ground-truth orientations with the different interpolation parameters, and examined the resulting tilt angles. A total of 139 reference diffraction patterns were created from −69° to +69° (which was the incident beam angle range used in the tomography experiment), with 1° tilt angle intervals with cubic spline interpolation. It should be noted that the angular range for reference diffraction patterns would not be limited in the case of single-particle XFEL data because these reference patterns would need to be computed in all orientations in such case. Two different matching regions were defined as earlier, *i.e.* one having *q*_L_ = 5 and *q*_H_ = 10 pixels and the other having *q*_L_ = 5 and *q*_H_ = 20 pixels. Fig. 6 ▸ shows the assigned tilt angles as a result of this test.

With the combination of η = 10 and α = 15, the tilt angles are not well estimated [Figs. 6(*a*) and 6(*b*) ▸], presumably because the reconstructed volume becomes too blurred as shown in Fig. 5(*d*) ▸. The use of large values of α compensates for this effect by emphasizing the nearby images for interpolation (Fig. 5*e* ▸). By increasing α to 100 with η = 10, the agreement between the estimated and ground-truth angles greatly improved [Figs. 6(*c*) and 6(*d*) ▸], and angles similar to ground-truth angles are obtained.

As we described in §2, we excluded the center region of the experimental diffraction patterns (*q* < 5 pixel) for CC calculations. The diffraction intensities in this region are very high and have large fluctuations such that the CC measure is much less discriminative if the pixels in this region are included in the calculations. The results with *q*_H_ = 10 were slightly better than those with *q*_H_ = 20 [Figs. 6(*c*) and 6(*d*) ▸]. The use of too large matching regions would not be beneficial since the pixels farther from the image center are more prone to noise. Therefore, all further experiments were performed using scaled cropped experimental diffraction patterns of size reduced to 50 pixel × 50 pixel, η = 10 pixel and α = 100 for volume reconstruction, and *q*_L_ = 5 pixel and *q*_H_ = 10 pixel for orientation estimation (these *q*_L_ and *q*_H_ correspond to 0.004 nm^−1^ and 0.002 nm^−1^ in reciprocal space, respectively).

### 3.4. Estimation of the incident beam angle from random initial reference volumes   

Using the parameters calibrated in the preliminary analysis presented above, we performed the tilt angle estimation using an initial reference volume computed from experimental images by assigning random orientations. We created five reference initial volumes using five sets of random angles (in the range [−69°, 69°]) for each experimental image. It should be again noted that there would not be such an angular range limit when using single-particle XFEL data (the initial volumes would be generated from experimental images at any random orientation in the case of single-particle XFEL data).

**Figure 7:**
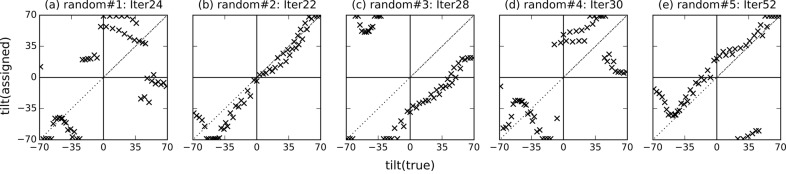
Tilt angles assigned to the experimental images by the proposed method initiated with five random initial volumes. The horizontal and vertical axes represent the ground-truth incident tilt angles and the tilt angles assigned by the proposed method, respectively. Iter is the number of iterations until convergence.

Fig. 7 ▸ shows the results of the tilt angle estimation. The parameters converged within 20 to 50 iterations. We obtained good results in two of five trials (random#2 and random#5), meaning that the estimated angles were similar to the ground-truth angles in these two trials. For the other three trials, although the estimated tilt angles largely deviated from the correct angles, the relative orientations of nearby diffraction patterns (the diffraction patterns with similar orientations) were partially recovered. For example, in the trial random#3, it appears as if [assigned 35, 70] should be wrapped to [−100, −70], but this is because this reconstructed volume is made from two disconnected groups of images; in one group, the images from [true −35, 70] are correctly aligned and in another the images from [true −65, −35] are aligned, but the connection around the image with [true −35] was not recovered during this iteration. Thus, two groups exchanged their positions in this iteration, and appear ‘swapped’. It should be noted, however, that such optimization issues may be less severe for data from a typical XFEL single-particle experiment. In such experiments, different pairs of diffraction patterns may share different common lines, unlike the single-tilt-axis tomographic CXDM data in which all patterns share just one common line. In the above particular example, if we had a few diffraction patterns that are (near) perpendicular to the tilt-axis, *i.e.* transverse to the other images, such remaining inconsistencies could be identified and possibly resolved. These results show that our 3D reconstruction method can successfully identify relative orientations of similarly oriented images although the results depend on their initial volume.

**Figure 8:**
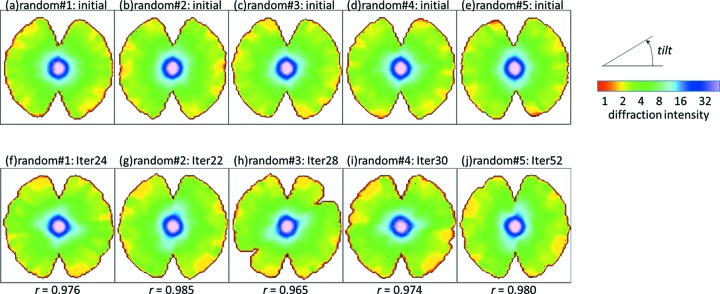
Cross section of (*a*–*e*) random initial volumes and (*f*–*j*) finally reconstructed volumes. The tilt axis is perpendicular to the cross section. The same cross-section view is shown for all volumes. The *r*-values shown below panels (*f*)–(*j*) are the correlation coefficients between finally reconstructed volumes and ground-truth volume (Fig. 5*e* ▸).

Fig. 8 ▸ shows the cross sections of the five initial random volumes and the corresponding final reconstructed 3D volumes. For all the trials, the cross sections of the initial and final volumes are significantly different. For the random#2 and random#5 trials, which showed good matching results in Fig. 7 ▸, final cross sections were similar to the cross sections of the volume reconstructed using the ground-truth angles and the same interpolation parameters (η = 10 pixel and α = 100) (Fig. 5*e* ▸). In the random#3 trial, slices were not assigned from 20° to 45° in both Fig. 7(*c*) ▸ and Fig. 8(*h*) ▸.

### 3.5. Cross-correlation map among diffraction patterns   

**Figure 9:**
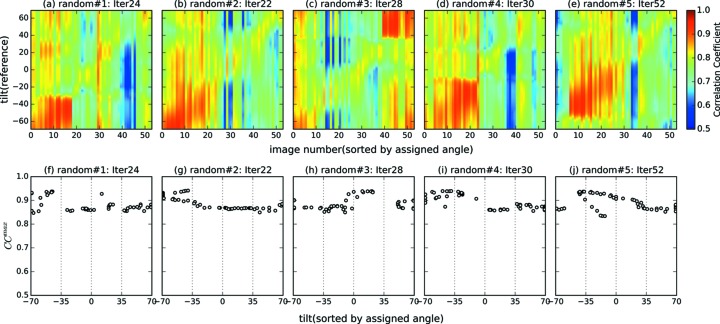
(*a*–*e*) CC map between the experimental images and the references created from the final volume. CC coefficients were calculated only for the matching regions (*q*_L_ = 5 pixels, *q*_H_ = 10 pixels). The horizontal axis represents the image numbers which were sorted by the assigned tilt angle. (*f*–*j*) Distribution of the assigned angle for each experimental image and the maximum CC coefficient (CC^max^) against the reference images created from the final model.

To further assess the results of the five trial runs, we examined the correlation coefficients between experimental images and all reference images which were sliced from the final volume. We created the cross-correlation map (CC map) as shown in Figs. 9(*a*)–9(*e*) ▸. In Figs. 9(*a*)–9(*e*) ▸, horizontal axes represent the image number, which were sorted by the assigned tilt angle at the end of the refinement to check the CC coefficient between assigned and all reference tilt angles. For the random#2 and random#5 trials, which showed good matching results, the maximum CC coefficient values mostly follow diagonal lines. This is more the case for random#2 than for random#5, which is consistent with the matching results as shown in Fig. 7 ▸. This means that the diffraction patterns are more smoothly arranged in the final volume for random#2 and random#5 than for other trials.

On the other hand, for unsuccessful reconstructions such as random#1, correlations between the experimental image and the reference slices are not continuously distributed. This is more evident in Figs. 9(*f*)–9(*j*) ▸, which show the maximum CC coefficient (CC^max^) between each experimental diffraction pattern and their optimal reference images from the final reconstruction. There are multiple gaps in random#1, random#3 and random#4, for example from −45° to −30°, from −20° to −10°, from 0° to 20° and from 25° to 35° in random#1, *i.e.* these tilt angles were not assigned in their final reconstruction. In some cases, multiple images are assigned to the same angles. For random#2, a set of images with ground-truth angles from −69° to −45° are assigned to the same angles (Fig. 9*g* ▸). A similar result was obtained for random#5 (from −45° to −20°) as shown in Fig. 9(*j*) ▸. This is likely to be due to similarities in the diffraction patterns at these angles. In addition, CC^max^ values are discontinuous before and after the gaps, indicating the fragmented matching. These results show that angular assignments are initial value dependent. Geometrical relations between similar images are identified by the algorithm and nearby images are sorted correctly; however, discontinuities in the image alignment occasionally occur, resulting in a set of independent blocks of correctly ordered images. However, these artifacts should be less significant for typical single-particle experiments as the rotation angles are expected to be distributed evenly.

## 4. Discussion   

**Figure 10:**
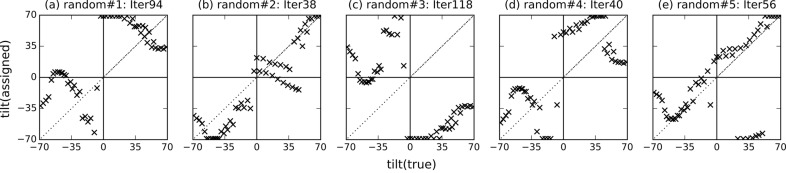
Tilt angles assigned to experimental images by reconstructing the diffraction intensity distribution (using slice matching and starting from the same random angles as in Fig. 7 ▸).

Our slice matching and 3D reconstruction protocol is similar to the cross-correlation maximization method previously developed by Tegze & Bortel (2012 ▸), in which a 3D reconstruction of NapAB protein molecule using a large number of simulated diffraction patterns (100000 images) was performed. In this study, we calculate the 3D structure factor amplitude distribution instead of the intensity distribution. Our tests have shown that matching results obtained using the 3D amplitude (Fig. 7 ▸) were slightly better than those obtained using the 3D intensity on the system studied here (Fig. 10 ▸). This may be attributed to the fact that the structure factor amplitude is smoother than the intensity because the square roots are taken. Also, we consider that it is more useful to reconstruct the 3D amplitude than the 3D intensity because the 3D amplitude can later be directly used to obtain the structure in real space. The results of our study show that such protocols can estimate the orientation of the incident beam angles and the 3D structure factor amplitude with experimental diffraction patterns.

Key parameters of our protocol are the parameters that determine the matching region (*q*_L_ and *q*_H_) and the interpolation parameters (η and α). Sometimes, these parameters are best adjusted by trial and error. We found that large *q*_L_ and small *q*_H_ values give optimal results since the experimental diffraction pattern pixels much further from the image center are unreliable due to noise and the central pixels have too strong intensities which reduce the discriminative power of the CC measure. It is also possible to use a low-wavenumber region first to determine the orientation of diffraction patterns to reconstruct the 3D structure factor amplitude coarsely, and later use a high-wavenumber region to determine the structure with finer details.

In addition, we carefully examined the choice of parameters for the interpolation with the Kaiser–Bessel window. In our test, we assembled the diffraction images of 50 pixel × 50 pixel (the wavenumber at the edge of the diffraction patterns is approximately 0.011 nm^−1^). Assuming that we evenly distribute 53 images over an angular range of 140°, the distance between the edge pixels on two adjacent images in 3D space is about 1 voxel. Using the combination of η = 10 and α = 100, the weight would decrease to a half value at the voxel about 1.5 units away from the position where the pixel on the 2D diffraction image is mapped within the 3D volume (Fig. 5*a* ▸). Thus, for each voxel, pixels from three to four images are weight-averaged. These parameter values are appropriate for 3D interpolation from a limited number of 2D diffraction patterns. We have found that the parameters η and α can be selected in a general case based on the rule that pixels from three to four images should be weight-averaged in 3D space.

We also showed that the reliability of the reconstructed volume could be examined by evaluating the continuity of CC^max^ with respect to the assigned beam angle [Figs. 9(*f*)–9(*j*) ▸]. Tegze & Bortel (2016 ▸) proposed an approach that evaluates the distribution of CC values between the diffraction patterns and the reference slices [like in Figs. 9(*a*)–9(*e*) ▸ of our study] assuming that just a few particular diffraction patterns should have high CC values. Our approach is complementary to this approach, focusing on the distribution of assigned angles and CC^max^ continuity.

In single-particle XFEL experiments, the signal-to-noise ratio could be improved by using a large number of diffraction patterns (by averaging many diffraction patterns with similar 3D orientations, as done in single-particle electron microscopy). However, this has not been demonstrated with experimental tomographic CXDM data because a small number of diffraction patterns is usually obtained. Also in our test study (with tomographic CDXM data), only 53 patterns were mapped onto a 140° angular space. Thus, the strategy to improve the signal-to-noise ratio with actual experimental data requires further study.

In addition, in this study, we simply assigned initial orientations randomly, because both the number of images and possibility of assignment angles were small. To create a reliable initial volume could be a challenging problem in single-particle analysis. However, Tegze & Bortel (2012 ▸) demonstrated encouraging results that the projection matching of NapAB protein starting from random orientations using 100000 diffraction patterns could converged after 15 iterations. Some optimization methods have been proposed in cryo-EM analysis (Vargas *et al.*, 2014 ▸; Sorzano *et al.*, 2015 ▸). These studies could help us to create an appropriate initial volume using XFEL diffraction images. By combining with a 3D classification for many final volumes obtained by a large number of trials, we can select the most populated classes as the most plausible volumes.

## 5. Conclusion   

We have developed a protocol for the 3D reconstruction of the absolute value of the structure factor from XFEL diffraction patterns and software based on *Xmipp* libraries to run it. In this article, we presented this protocol and the results of its tests with experimental diffraction patterns of an aerosol nanoparticle obtained by CXDM. Although the angle estimation for these data is difficult, as only one common line exists between diffraction patterns, encouraging results were obtained. Reconstruction was performed starting from randomly created reference 3D structures, and nearly correct volumes were obtained. We also showed that the plausibility of reconstructed volumes could be evaluated by examining the continuity of CC^max^ with respect to the assigned beam angles. Finally, we showed the sensitivity of the protocol to different parameter values. Additional testing with more experimental data would be necessary to establish more general guidelines to the parameter adjustment.
